# Genetic differentiation in pesticide resistance between urban and rural populations of a nontarget freshwater keystone interactor, *Daphnia magna*

**DOI:** 10.1111/eva.13293

**Published:** 2021-10-01

**Authors:** Kristien I. Brans, Rafaela A. Almeida, Maxime Fajgenblat

**Affiliations:** ^1^ Laboratory of Aquatic Ecology, Evolution, and Conservation KU Leuven Leuven Belgium

**Keywords:** chlorpyrifos, *Daphnia magna*, organophosphate pesticides, pesticide resistance, urban evolution

## Abstract

There is growing evidence that urbanization drives adaptive evolution in response to thermal gradients. One such example is documented in the water flea *Daphnia magna*. However, organisms residing in urban lentic ecosystems are increasingly exposed to chemical pollutants such as pesticides through run‐off and aerial transportation. The extent to which urbanization drives the evolution of pesticide resistance in aquatic organisms and whether this is impacted by warming and thermal adaptation remains limitedly studied. We performed a common garden rearing experiment using multiple clonal lineages originating from five replicated urban and rural *D*. *magna* populations, in which we implemented an acute toxicity test exposing neonates (<24h) to either a solvent control or the organophosphate pesticide chlorpyrifos. Pesticide exposures were performed at two temperatures (20°C vs. 24°C) to test for temperature‐associated differences in urbanization‐driven evolved pesticide resistance. We identified a strong overall effect of pesticide exposure on *Daphnia* survival probability (−72.8 percentage points). However, urban *Daphnia* genotypes showed higher survival probabilities compared to rural ones in the presence of chlorpyrifos (+29.7 percentage points). Our experiment did not reveal strong temperature x pesticide or temperature x pesticide x urbanization background effects on survival probability. The here observed evolution of resistance to an organophosphate pesticide is a first indication *Daphnia* likely also adapts to pesticide pollution in urban areas. Increased pesticide resistance could facilitate their population persistence in urban ponds, and feed back to ecosystem functions, such as top‐down control of algae. In addition, adaptive evolution of nontarget organisms to pest control strategies and occupational pesticide use may modulate how pesticide applications affect genetic and species diversity in urban areas.

## 1 INTRODUCTION

Humans ubiquitously impact natural ecosystems in various ways (Hendry et al., 2017; Palumbi, 2001). Strong forms of human‐driven land conversion and habitat and microclimate alterations are concerted in cities (Alberti et al., 2020; Grimm et al., 2008; Parris, 2016); urbanization modifies the physical, chemical, and biological characteristics of natural habitats, structurally and functionally changes connectivity patterns among biological communities, induces disturbances of which many are novel (e.g. chemical contaminants and noise), and finally alters biotic interactions as a consequence of changes in native species composition and the introduction of non‐native species. The impact of urbanization on species distributions and diversity, community assembly, and interspecific trait variation has been extensively monitored (Aronson et al., 2014, 2016; Dallimer et al., 2012; Merckx et al., 2018; Piano et al., 2017). In recent years, it has become clear that cities moreover drive contemporary evolution (Donihue & Lambert, 2015; Johnson & Munshi‐South, 2017). A large number of observations of urban evolution focuses on neutral evolution stemming from the impact of habitat fragmentation, among‐city connectivity hubs, and population size changes on gene flow and genetic drift (Miles et al., 2019; Munshi‐South & Richardson, 2020). In addition, evidence on adaptive evolution in response to urban temperature gradients (e.g. acorn ants: Diamond et al., 2017; Martin et al., 2019; water fleas: Brans et al., 2018; Brans, Jansen, et al., 2017), fragmentation (e.g. holy hawksbeard: Cheptou et al., 2017), and specific chemical pollution (e.g. polychlorinated biphenyl resistance in tomcod: Wirgin et al., 2011; and killifish: Oziolor & Matson, 2018; Whitehead et al., 2012) is growing incessantly.

One example of urban adaptation is documented in the water flea *Daphnia magna* (Brans & De Meester, 2018; Brans, Engelen, et al., 2018; Brans, Govaert, et al., 2017; Brans, Jansen, et al., 2017). *Daphnia magna*, a large‐bodied cladoceran, resides in lakes and ponds, and is an important interactor in the freshwater aquatic food web; *Daphnia* exerts top‐down control on algae and can prevent the occurrence of nuisance algal and bacterial blooms as a consequence of its size‐related grazing efficiency (Chislock et al., 2013; Gianuca et al., 2016). Compared to rural populations, urban populations of water fleas show an evolved higher heat tolerance (Brans, Jansen, et al., 2017), faster pace‐of‐life (Brans & De Meester, 2018), altered stress physiology and a higher energy budget (Brans, Engelen, et al., 2018). Urban ponds in the study region (Flanders, Belgium) are significantly warmer compared to rural ponds (differences in summer mean and maximum temperature range between 3°C–4°C; Brans, 2018), driving observed genetic increases in heat tolerance, haemoglobin concentrations, as well as body size reductions (Brans, Jansen, et al., 2017). While evidence on thermal adaptation in urban water fleas is profound, to what extent they adapt to pollution in urban areas, and whether interactions between warming and pollution have effects on biotic evolutionary and ecological responses (Cuenca Cambronero et al., 2018; Moe et al., 2013; Noyes et al., 2009; Theys et al., 2020) is not known in this study system.

Residing in closed ecosystems such as ponds and pools, aquatic biota are exposed to a multitude of stressors, including chemical contaminants such as pesticides (Allinson et al., 2015; Meftaul et al., 2020). In cities, pesticides enter freshwater ecosystems via run‐off and aerial transportation (Allinson et al., 2015; Meftaul et al., 2020; Wittmer et al., 2010), after application in public places, such as parks and schools, or for occupational purposes (e.g. domestic indoor use, private gardens) (Meftaul et al., 2020; Pang et al., 2002). After prohibition of organochlorines (e.g. DTT) in the 1970s, pyrethroids and organophosphate pesticides (e.g. chlorpyrifos, glyphosate, malathion, diazinon and carbaryl) were extensively and commonly used to suppress nuisance pests (insects, fungi) and unwanted weeds in municipal, business and domestic environments (Heudorf et al., 2004; Julien et al., 2008; McKelvey et al., 2013; Vijftigschildt et al., 2005; Wittmer et al., 2010). Such pesticide exposures in combination with warming (as a consequence of urban heat islands) in cities could synergistically alter evolutionary and ecological responses in *Daphnia*. For example, warming and heat stress could increase the toxicity of a pesticide, directly or via the increased uptake of the toxicant as a consequence of altered metabolic processes and, vice versa, toxicant exposure could limit acclimation or adaptation to warming and heat stress (Holmstrup et al., 2010; Hooper et al., 2013; Moe et al., 2013; Noyes et al., 2009; van den Brink et al., 2018).

*Daphnia* is a well‐established ecotoxicological model system in ecological risk assessment of pesticides by means of standardized acute and chronic toxicity testing (Brock & Van Wijngaarden, 2012; Huang et al., 2020; Palma et al., 2008). Organophosphate pesticides impact *Daphnia* survival, embryological development, fertility, endocrine function, life history changes, and stress physiological parameters such as enzymatic defence and energy budget (Ferrario et al., 2018; Jeon et al., 2013; Palma et al., 2009; Pérez et al., 2015; Silva et al., 2018; Song et al., 2017; Wang et al., 2009; Zalizniak & Nugegoda, 2006). Impacts tested in more realistic settings often focus on the assessment of relative sensitivities of multiple organism groups (Cuppen et al., 2002; Echeverri‐Jaramillo et al., 2020; Gaizick et al., 2001; Pérez et al., 2015), the impact of individual interacting environmental variables on model organisms (e.g. food concentration, shelter availability; Augusiak & van den Brink, 2016), the toxicity of pesticide mixtures, drainage ditch water or rain water samples on nontarget species (Cáceres et al., 2007; Hasenbein et al., 2017; Sakai, 2002; Wood & Stark, 2002) or ecosystem level interactions in standardized indoor or outdoor mesocosms (Kersting & van Wijngaarden, 1992; Knillmann et al., 2012; López‐Mancisidor et al., 2008; van den Brink et al., 1995; Zafar et al., 2011). While such experiments are crucial to improve ecological risk assessment of pesticides and the mechanistic understanding of their specific modes of action and detrimental effects, they often overlook the possibility that populations can evolve resistance to applied toxicants (e.g. using ‘naïve’ pooled populations and communities of zooplankton, Rumschlag et al., 2020). Evolved pesticide resistance in natural populations of *Daphnia* in response to agricultural pesticide application was shown both across spatial gradients (organophosphates: Bendis & Relyea, 2014; carbamates: Coors et al., 2009; Jansen et al., 2015) as well as in response to historical contamination (resurrection ecology, organophosphates: Simpson et al., 2017). Such adaptive evolutionary responses can have ecosystem‐wide buffering effects (e.g. shown in *D*. *pulex*: Bendis & Relyea, 2016a, 2016b) and could potentially occur in cities, both in response to pesticide pollution, warming, and a combination of both.

Studies on evolved pesticide resistance in urban areas are so far focused on disease vectors and pests (Zhu et al., 2016) such as malaria and dengue transmitting mosquitos (Kamdem et al., 2017; Li et al., 2018; Macoris et al., 2003; Pereira Lima et al., 2003), bed bugs (Romero & Anderson, 2016), and cockroaches (Wu & Appel, 2017). Nevertheless, nontarget organisms, especially those residing in closed aquatic systems, such as ponds and pools, could also evolve pesticide resistance. For example, exposure to contaminated water way run‐off leads to pyrethroid resistance in freshwater crustaceans (amphipods; Major et al., 2018). So far, only one specific example is available on the evolution of resistance to pesticides in nontarget species in a multi‐stressor urban context (warming x pesticide exposure) (Tüzün et al., 2015); using a common garden approach, urban and rural damselfly nymphs (*Coenagrion puella*) were exposed to sublethal concentrations of chlorpyrifos at two different temperatures (20°C vs. 24°C—mimicking observed urban heat islands in the study region, Flanders, air: Wouters et al., 2017, water: Brans, Engelen, et al., 2018). Upon exposure to chlorpyrifos, urban genotypes increase their activity and keep food intake constant at both 20°C and 24°C, in contrast to rural genotypes, which show a reduction in both behavioural parameters at both temperatures. The development of such pesticide resistance in nontarget organisms across multiple trophic levels could modulate genetic and species diversity, as well as ecosystem functions and services in urban areas (Bendis & Relyea, 2016b; Des Roches et al., 2020).

This study primarily aims to explore whether urban *D*. *magna* evolved resistance to pesticides and to what extent responses are magnified by warming (Knillmann et al., 2013; Moe et al., 2013; Theys et al., 2020) and/or shaped by thermal adaptation (Op de Beeck et al., 2017; Dinh Van et al., 2013). We performed a common garden rearing experiment using replicated urban and rural populations of *Daphnia magna*. We assessed survival after an acute exposure (48h) to the model organophosphate pesticide chlorpyrifos, at two different temperatures, 20°C and 24°C (mimicking the urban heat island effect observed in ponds in the study region, Brans, Engelen, et al., 2018). Chlorpyrifos is a broad‐spectrum organophosphate insecticide that inhibits acetylcholinesterase (Giddings et al., 2014) and has been applied in urban areas worldwide (Heudorf et al., 2004; McKelvey et al., 2013; Vijftigschildt et al., 2005). It is considered to be a priority pollutant in the European Water Framework as per Directive 2000/60/CE. Chlorpyrifos has been shown to induce signals of adaptive phenotypic trait change in urban damselflies in the same study region (Flanders, Belgium) and was not yet prohibited during the time period of sampling populations of *D*. *magna* and *Coenagrion puella* (2013–2015; Speedy sampling, see also Merckx et al., 2018). We hypothesize that (1) urban genotypes will have a higher survival when exposed to chlorpyrifos as a consequence of evolved resistance to organophosphates, (2) survival in response to chlorpyrifos might be temperature‐dependent due to a higher toxicity of chlorpyrifos at higher temperatures, and (3) temperature‐dependent resistance responses differ between urban and rural genotype sets as a consequence of thermal adaptation in urban populations.

## 2 MATERIAL AND METHODS

### 2.1 Study populations, experimental design and pre‐experimental conditions

**FIGURE 1:**
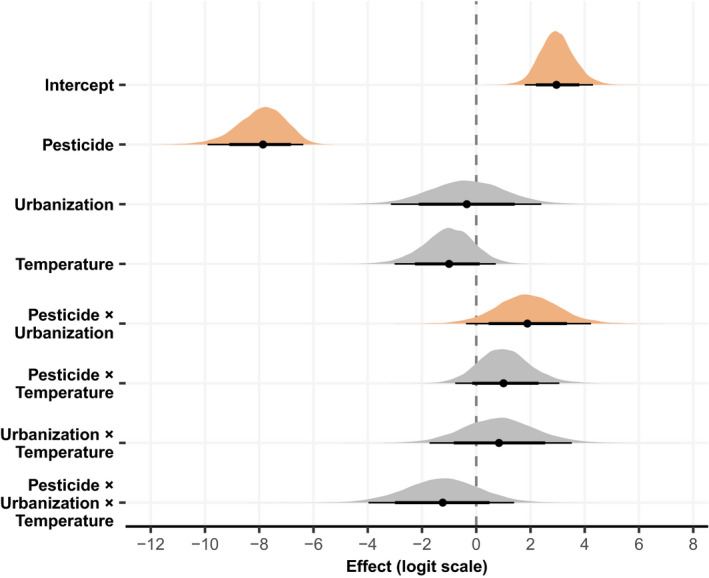
Estimated main and interaction effects on survival (logit scale). Bayesian posterior densities are represented by density plots, 80% and 95% credible intervals by horizontal lines, and posterior medians by black dots. Negative values correspond to a decreased survival probability, while positive values correspond to an increased survival probability, relative to the average condition, population and clone. Variables for which the posterior probability that their effect is either positive or negative exceeds 0.95 (given model and data) are shown in orange

*Daphnia magna* populations originated from five urban and five rural populations (Table S1) located in Flanders (Belgium) and are a subset of the 13 populations used in Brans, Jansen, et al. (2017) for which clonal lineages have been established in the laboratory (2012–2013, Speedy sampling campaign (Brans, Govaert, et al., 2017; Brans, Jansen, et al., 2017; Merckx et al., 2018). Flanders is a densely population region (ca. 371, amounting to 693 inhabitants/km^2^, IBZ, 2021). Urbanization levels were assessed based on the percentage built‐up area (BA) in the regional surroundings of the pond (3200 m radius as in Brans & De Meester, 2018; Brans, Govaert, et al., 2017; Brans, Jansen, et al., 2017; Brans et al., 2018), based on the Large‐scale Reference Database (LRD, 2013). Urban populations are characterized by >10% BA and nonurban (‘rural’) populations <5% (Table S1; range urban: 14.12%–40.44% BA; range rural: 0.63%–4.38%; see also Figure 1 in Brans & De Meester, 2018). Percentage BA does not include roads and parking lots, which results in ponds characterized by more than 10%–15% of BA or higher (in this case all urban ponds >14% BA) being already substantially urbanized (Brans & De Meester, 2018). Percentage BA has shown to be a reliable proxy of urbanization as it, in the specific study region, positively correlates with impervious substrate cover like roads and artificial constructions such as viaducts bridges, and is negatively associated with the amount (area) of semi‐natural habitat (Piano et al., 2020). In addition, rural nonurban locations had to meet the criterion of minimum 20% biologically valuable area (based on the Biological Value Map Flanders that includes land cover and vegetation mapping according to a biological valuation based on ecological criteria such as biodiversity, rarity, vulnerability and replaceability of the biotope; Brans, Govaert, et al., 2017; Saeger et al., 2017) in their regional surroundings (3200 m radius) to prevent sampling populations in nonurban agricultural areas.

Individuals from the five clonal lines from each population (*n* = 50) were inoculated in 200‐mL glass jars filled with dechlorinated tap water, reared under standardized laboratory conditions (20°C, 16:8 L:D photoperiod) and fed *ad libitum* with the unicellular green algae *Acutodesmus obliquus* (1 × 10^5^ cells/mL, coinciding with the Incipient Limiting Level of 1 mg C/L). 80% of the medium was refreshed every other day. After the first generation, lineages were split into eight independent culturing units, using parthenogenetic offspring of mother individuals, in order to generate replicated lines that could later be exposed to the pesticide treatment (control/chlorpyrifos) and temperature treatment (20°C/24°C) in a full factorial crossing. Each clone × temperature × pesticide treatment combination was replicated twice. To obviate interference from (grand)maternal effects, these eight clonal replicates were cultured for a minimum of two consecutive generations before pesticide exposures took place. Newborn juveniles were eliminated to prevent overcrowding. Second to fourth clutch juveniles were used to start up new generations or to start up the experimental exposure phase (see further). This overall design resulted in 2 urbanization classes (rural/urban) × 5 populations × 5 clones × 2 pesticide treatments (control/pesticide) × 2 temperatures (20°C/24°C) × 2 replicates = 400 experimental units. Due to the continuous infestation of one clone (u2.5) with a parasitic infection and consequently weak offspring production, and eventually mortality, it was completely excluded from the experiment (resulting in a total number of 392 experimental units).

### 2.2 Pesticide exposure and survival

We exposed five juveniles (<24h old but older than 5h) per experimental unit according to OECD guidelines (2004) to either a control (solvent) or chlorpyrifos (CAS: 2921‐88‐2, purity >99%, Sigma‐Aldrich; nominal concentration of 0.67 µg/L), at both 20°C and 24°C, using 100‐mL glass recipients filled with 50 mL of the exposure medium (control medium/pesticide medium—no feeding, OECD, 2004). The pesticide exposure medium was prepared daily from a stock solution (1 mg/mL, Sigma‐Aldrich Chlorpyrifos powder >99% purity dissolved in absolute ethanol (100%), stored in an dark brown glass bottle and kept at 4°C in dark conditions). The stock solution was diluted first with Mili‐Q (step 1, intermediate stock) and secondly with dechlorinated tap water (step 2, exposure medium) to achieve the nominal concentration of 0.67 µg/L in the exposure medium. For the solvent control medium the same amount of absolute ethanol (100%) was used in the first dilution step (instead of the pesticide). The nominal 0.67 µg/L chlorpyrifos concentration was set after an initial range finding pilot exposure with nominal chlorpyrifos concentrations ranging between 0.3 and 0.85 µg/L, using a pooled batch of animals (urban and rural mixed). The chosen concentration lies between the range for which EC50 values of *D*. *magna* have been found (0.6 µg/L in Moore et al., 1998; 0.25 µg/L in Naddy et al., 2009; 0.74 µg/L in Palma et al., 2008; 0.49 µg/L in Raymundo et al., 2019) and within the observed range of concentrations found in European surface waters in the wider region (waters: 95% confidence interval = [0.07, 0.69 μg/L]) (Schulz, 2004; Stehle & Schulz, 2015). Immobilization was assessed after 24 and 48h (OECD, 2004), with the criterion of minimally antennae movement to be clearly observed. Upon counting immobilized animals, we noticed six instead of five juveniles to be present in four (out of 392) experimental units. Given we follow OECD acute toxicity assay guidelines, which require pesticide exposures to be performed in absence of food, we confirmed the potential impact (e.g. differences in energy acquisition) of this manual error to be minimal (see further). In addition, newborn juveniles (<24h old) perform limited to no grazing activity (Cowgill et al., 1984).

### 2.3 Statistical analyses

We use a Bayesian binomial generalized linear mixed model to estimate the effect of pesticide treatment (control/pesticide), urbanization background (rural/urban) and temperature (20°C/24°C), as well as their interactions, on *D*. *magna* survival probability after 48h. More specifically, we assume that the number of surviving individuals $y_{i}$ of the i’th replicate follows a binomial distribution:

$$y_{i}∼\mathrm{Binomial}\left(N_{i},p_{i}\right),$$

where $N_{i}$ is the replicate's initial number of individuals (5, or exceptionally 6), and $p_{i}$ is the individual survival probability, modelled through the following logit link function:

$$\log\left(\frac{p_{i}}{1‐p_{i}}\right)=\beta_{0}+{\mathrm{CPF}}_{i}∙\beta_{\mathrm{CPF}}+{\mathrm{URB}}_{i}∙\beta_{\mathrm{URB}}+T_{i}∙\beta_{T}+{\mathrm{CPF}}_{i}∙{\mathrm{URB}}_{i}∙\beta_{CPF:URB}+{\mathrm{CPF}}_{i}∙T_{i}∙\beta_{CPF:T}+{\mathrm{URB}}_{i}∙T_{i}∙\beta_{URB:T}+{\mathrm{CPF}}_{i}∙{\mathrm{URB}}_{i}∙T_{i}∙\beta_{CPF:URB:T}+{\mathrm{CPF}}_{i}∙b_{pop\left(i\right)}+{\mathrm{CPF}}_{i}∙b_{clone\left(i\right)},$$

where $\beta_{0}$ is the intercept, ${\mathrm{CPF}}_{i}$ indicates whether the individuals of the i’th replicate were exposed to chlorpyrifos (0.5) or not (−0.5), ${\mathrm{URB}}_{i}$ indicates whether the individuals of the i’th originated from a rural (−0.5) or urban (0.5) population,$T_{i}$ indicates whether the individuals of the i’th replicate were exposed to 20°C (−0.5) or 24°C (0.5), the $\beta_{∙}$‐parameters represent the corresponding main and interaction effects, and $b_{pop\left(i\right)}$ and $b_{clone(i)}$ are the population‐ and clone‐level random effects corresponding to the i’th replicate. As the input variables CPF, URB and T are centred around zero, the intercept $\beta_{0}$ represents the average condition.

Heterogeneity among populations with respect to sensitivity to chlorpyrifos exposure is modelled through a normally distributed population‐level random effect $b_{\mathrm{pop}}$:

$$b_{\mathrm{pop}}∼\mathrm{Normal}\left(0,\sigma_{\mathrm{pop}}\right),$$

where $\sigma_{\mathrm{pop}}$ is the population‐level standard deviation. Remaining heterogeneity among clones that is not explained by the population‐level random effects is modelled by means of a clone‐level random effect $b_{\mathrm{clone}}$. As a preliminary exploration revealed some presumably outlying clone‐specific responses, we model the clone‐level random effects with a Student's *t*‐distribution:

$$b_{\mathrm{clone}}∼\mathrm{StudentT}\left(\nu ,0,\sigma_{\mathrm{clone}}\right),$$

where ν is the number of degrees of freedom, and $\sigma_{\mathrm{clone}}$ is the clone‐level scale parameter (which we call standard deviation below for simplicity). For low values of ν, the Student's *t*‐distribution has substantially fatter tails compared to the normal distribution, offering robustness against unusual observations. As ν→∞, the Student's *t*‐distribution approximates the normal distribution. By treating ν as an unknown quantity (to be estimated as part of the model), the thickness of the distribution's tail is tuned according to the data and the prior (Gelman et al., 2014).

We choose weakly informative priors for all model parameters:

$$\beta ∼\mathrm{StudentT}\left(3,0,5\right),$$

$$\sigma_{\mathrm{pop}},\sigma_{\mathrm{clone}}∼{\mathrm{StudentT}}^{+}\left(3,0,5\right),$$

$$\nu ∼\mathrm{Gamma}\left(2,0.1\right).$$

As the priors on β (the vector of all regression coefficients), $\sigma_{\mathrm{pop}}$ and $\sigma_{\mathrm{clone}}$ place most prior mass around zero, they mainly have a regularizing effect on the posteriors distribution (i.e. shrinking estimates towards 0 unless the data indicates otherwise). The Gamma‐prior on ν is chosen following the recommendations by Juárez and Steel (2010).

We implemented this Bayesian robust generalized linear mixed model using the probabilistic programming language Stan and performed Markov chain Monte Carlo (MCMC) sampling through the rstan v.2.21.2 package (Stan Development Team, 2020) in R v.4.0.3 (R Core Team, 2020). Stan performs Bayesian inference by means of dynamic Hamiltonian Monte Carlo (HMC), a gradient‐based MCMC sampler (Carpenter et al., 2017). We ran four chains with 10,000 iterations each, of which the first 5,000 were discarded as warmup. The resulting 20,000 posterior samples are summarized using posterior medians and 95% equal‐tailed credible intervals (bounded by the 2.5% and 97.5% samples from the distribution), unless otherwise specified. We used the tidybayes v.2.3.1 package (Kay, 2020) to visualize the posterior distributions.

We assessed model convergence both visually by means of traceplots and numerically by means of effective sample sizes, divergent transitions and the Potential Scale Reduction Factor, for which all parameters had $\hat{R}<1.01$ (Vehtari et al., 2019). We performed posterior predictive checks to assess goodness‐of‐fit and performed a sensitivity analysis to assess the influence of the chosen priors (see Supporting Information, Figures S1–S13).

The full code for the analyses is available through https://github.com/mfajgenblat/brglmm‐chlorpyrifos.

As mentioned, there were four experimental units which contained six instead of five juveniles. Analyses of the data excluding these four observations did not qualitatively change the results (see further and Figure S14, Supplementary Information).

## 3 RESULTS

**FIGURE 2:**
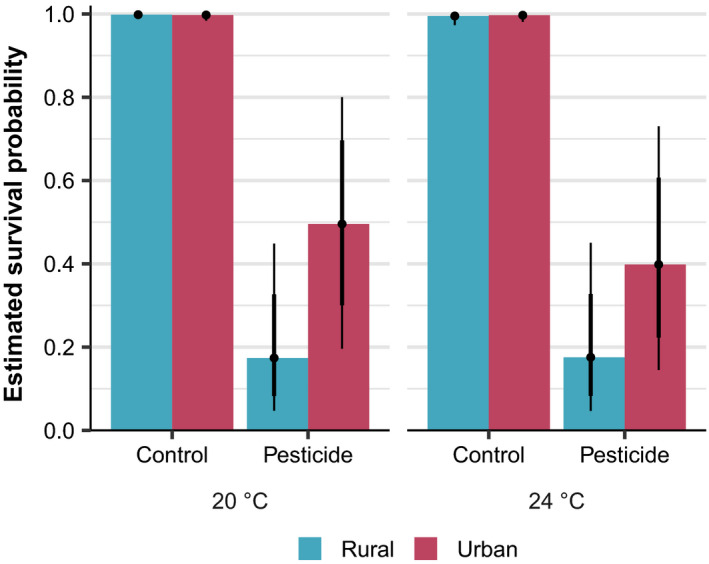
Additive visualization of Figure 1: estimated survival probability after 48h for each combination of urbanization level (rural, blue vs. urban, red), chlorpyrifos exposure treatment (control vs. pesticide), and temperature treatment (20°C vs. 24°C). 80% and 95% credible intervals are shown by vertical lines and posterior medians by black dots

In this experiment, *Daphnia magna* individuals faced important reductions in survival probability at 48h upon exposure to chlorpyrifos, with an estimated effect of −7.86 (95% CrI [−9.93, −6.37]) on the logit scale (Figure 1). On the probability scale, the marginal effect (at the mean values of the other variables) of chlorpyrifos exposure corresponds to a reduction of 72.80 percentage points (95% CrI [0.50, 0.88]) in survival probability: survival is 99.90% (95% CrI [99.42, 99.99]) without exposure and drops to 27.07% (95% CrI [11.66, 49.70]) with exposure (Figures 1, 2). By contrast, the main effects of urbanization and temperature on survival probability are estimated to be much weaker, respectively −0.35 (95% CrI [−3.16, 2.42]) and −1.01 (95% CrI [−3.03, 0.70]) (Figure 1). On the probability scale, an urban population origin and a 24°C temperature confers a marginal reduction in survival probability of, respectively 1.41 (95% CrI [−20.29, 18.02]) and 4.43 (95% CrI [−5.20, 15.30]) percentage points, at the mean values of the other variables.

However, we identified a positive interaction effect between chlorpyrifos exposure and urbanization, with 95.16% posterior probability (Figure 1). This effect is estimated to be 1.91 (95% CrI [−0.38, 4.22]) on the logit scale. Equivalently, urban *Daphnia* individuals exposed to chlorpyrifos have a 44.48% (95% CrI [16.71, 75.90]) marginal probability of survival, compared to 14.76% (95% CrI [3.78, 40.66]) for rural individuals, considering the mean values of the other variables (Figures 1, 2). The interactions effects between chlorpyrifos exposure and temperature and between urbanization and temperature are estimated to be smaller; 1.02 (95% CrI [−0.75, 3.06]) and 0.84 (95% CrI [−1.75, 3.52]) on the logit scale, respectively (Figure 1).

The three‐way interaction between chlorpyrifos exposure, urbanization and temperature is estimated to be negative with 81.73% posterior probability and has an estimated effect of −1.24 (95% CrI [−4.01, 1.40]) on the logit scale (Figure 1). The estimated survival probabilities for each of the eight considered treatment combinations are graphically summarized in Figure 2.

Furthermore, we identified important heterogeneity with respect to chlorpyrifos sensitivity among populations and clones, which was expected as we included replicated urban and rural populations and clones. The estimated posterior medians for the population‐ and clone‐level standard deviations equal 1.25 (95% CrI [0.59, 2.60]) and 1.12 (95% CrI [0.73, 1.58]), respectively. The estimated random effects for all clones, corrected for urbanization level (through the fixed effects), are visualized in Figure S15 (Supplementary Information) and show some particularly striking deviations compared to other clones in one rural and one urban population. These findings indicate that within populations, among‐clonal differentiation in tolerance responses to the pesticide can be found, sometimes to important extents, and thus reflect evolutionary potential. This is a common observation in *Daphnia* and in concordance with studies on thermal adaptation in response to urban heat islands and climate change (Brans, Jansen, et al., 2017; Geerts et al., 2015). The use of a robust Student's *t*‐distribution random effects specification accommodates these outlying observations by preventing a strong influence on the location and scale parameter estimates. Indeed, the estimated number of degrees of freedom for the Student's *t*‐distribution used for the clone‐level random effects equals 14.76 (95% CrI [2.83, 53.64]), corresponding to a distribution with somewhat fatter tails than the normal distribution.

## 4 DISCUSSION

In general, *Daphnia* survival was impaired by the acute exposure (48h) to the organophosphate pesticide chlorpyrifos compared to the control (−72.8 percentage points). However, we found strong evidence for a pesticide x urbanization effect, reflecting that urban water fleas showed a higher survival probability (+29.7 percentage points) compared to their rural counterparts in the presence of chlorpyrifos. While survival in response to the chlorpyrifos treatment was strongly impacted by the evolutionary urbanization background of the populations, we found no support for urbanization‐driven survival differences at different exposure temperatures (20°C vs. 24°C). We here discuss the observed effects in more detail.

*Daphnia* survival probability after acute exposure (OECD, 2004) was strongly impacted by chlorpyrifos at the used nominal test concentration of 0.67 µg/L (−72.8 percentage points, Figure 1). This is in concordance with previous observations of the range of chlorpyrifos concentrations for which EC50 values in *Daphia* have been reported (Huang et al., 2020; Palma et al., 2008). Importantly, we found that urban *Daphnia* genotypes had a higher survival probability when exposed to chlorpyrifos compared to rural genotypes (Figures 1 and 2). As expected, survival in the control was high for both rural and urban genotype sets (95.8% and 94.2%, respectively), without support for any important difference. However, animals originating from rural ponds show an expected drop of 29.7 percentage points in survival probability in the presence of the organophosphate pesticide compared to urban animals (from 44.48% marginal survival probability in urban to 14.76% in rural). These results confirm the first hypothesis and indicate evolved pesticide resistance in urban water fleas. The present observation supports studies that show species in urban areas can evolve resistance to pesticide applications (Zhu et al., 2016), mostly confirmed for target species (Li et al., 2018; Macoris et al., 2003; Romero & Anderson, 2016). For example, mosquitos of the *Anopheles gambiae* complex (malaria vectors) in urban areas developed resistance to insecticides (Kamdem et al., 2017), which was supported by genome scans showing selective sweeps on genes whose functions include epidermal growth, olfaction, cuticle formation, and resistance and detoxification of insecticides (Kamdem et al., 2017). More importantly, our study exemplifies the evolutionary impacts of pesticide applications on nontarget species in cities, which was so far limitedly demonstrated. The present results correspond strikingly to the urbanization‐driven evolution of organophosphate resistance in damselfly nymphs sampled in the same region (Flanders, Belgium—2013–2015) and exposed to the same pesticide (Tüzün et al., 2015). In a common garden experiment with F0 nymphal stages, Tüzün et al. (2015) demonstrated that urban damselfly nymphs increase activity and maintain feeding behaviour under chronic exposure to chlorpyrifos (2 µg/L), compared to rural damselfly nymphs. While different in set‐up (testing sublethal concentrations in a chronic exposure vs. acute toxicity testing, damselfly nymphs vs. water fleas, respectively), both studies build up the evidence for urbanization‐driven evolution of organophosphate resistance in aquatic nontarget species in Flanders (Belgium). Importantly, concentrations applied in Tüzün et al. (2015) and our study highlight a drastic difference in species sensitivity to organophosphate pollution (specifically chlorpyrifos in both cases) across interacting species (damselfly nymphs are common predators of water fleas). A contamination of urban waters at concentrations nonlethal to damselfly nymphs would imply already drastic mortality in one of their important prey components, which could potentially have indirect effects on biotic responses to pesticides observed in urban damselflies. We therefore advocate the need to perform experiments on multiple (interacting) species across food webs, both in ecotoxicological studies and in studies that aim to disentangle and understand eco‐evolutionary feedbacks in response to anthropogenic disturbances, such as urbanization (De Meester et al., 2019).

Though speculative, we present a number of potential mechanisms based on literature that could underlie the genetic differentiation in pesticide resistance between urban and rural *Daphnia magna* genotypes and that need further investigation in future research. Urban water fleas from the same urban study gradient evolved a higher energy budget, reflected in higher concentrations of protein, fat and carbohydrates in their body tissue (Brans, Stoks, et al., 2018). Higher net levels of energy compounds can enable an increased energy allocation to detoxification mechanisms under stress (De Coen & Janssen, 2003; Sokolova, 2013). Short‐term exposure of *Daphnia* to pesticides and metals indeed induces significant depletions in energy compounds (De Coen & Janssen, 2003; Sancho et al., 2009), and thus, evolved higher constitute levels of such compounds could be an adaptive buffer mechanism for toxicant adaptation (Brans, Stoks, et al., 2018). Given their high evolutionary potential and shown adaptations to a variety of anthropogenic stressors (Coors et al., 2009; Geerts et al., 2015; Jansen et al., 2015; Messiaen et al., 2010), selection on genes involved in detoxification and cellular protection, as observed in mosquitos (Kamdem et al., 2017), could occur in urban water fleas, but needs verification via genomic approaches.

We found no strong evidence for temperature‐mediated responses to the pesticide exposure such as warming‐induced toxicant sensitivity (Moe et al., 2013; Noyes et al., 2009). First, *Daphnia* survival probability when exposed to chlorpyrifos did not differ between 20°C and 24°C (pesticide × temperature, Figure 1) and is thus not in line with our second hypothesis. Warming‐induced increased toxicant sensitivity has been reported for chlorpyrifos before, including exposures to increases in mean temperature and temperature variation (freshwater isopods: Theys et al., 2020; *Daphnia*: Barbosa et al., 2017; Cuenca Cambronero et al., 2018). While we applied a 4°C temperature increase, it is argued the combination of both mean temperatures combined with daily temperature variation will more strongly impact biotic responses to pesticides, as organisms experience more extreme temperatures and thus also are challenged at more extreme physiological limits (Verheyen et al., 2019). In addition, while urban water flea genotypes have a higher pesticide resistance compared to rural genotypes, the data do not strongly support an urbanization × pesticide × temperature response (Figure 1), similarly as reported for damselfly nymphs (Tüzün et al., 2015). Urban water fleas are exposed to higher mean temperatures in urban ponds (Brans, Engelen, et al., 2018) and show signals of urban thermal adaptation (increased heat tolerance and haemoglobin concentrations, smaller body size) (Brans, Jansen, et al., 2017). We expected that chlorpyrifos resistance in urban *Daphnia* would be higher at 24°C compared to resistance in rural animals at that temperature, given thermal adaptation can offset pesticide toxicity under warming conditions (Op de Beeck et al., 2017; Dinh Van et al., 2013). Possibly, such responses would be more strongly elicited under more stressful thermal conditions such as heat spikes or heatwaves (Tüzün & Stoks, 2020). We did not apply a heat spike or heatwave in our experimental design as the exposure would need to last up to a minimum of five days (heatwave), which does not conform OECD (2004) guidelines of an acute toxicity test (48–72h). We propose future research to include a longer sublethal exposure to chlorpyrifos in combination with the occurrence of a heatwave, after which the organisms can be monitored for life history and physiological (energy budget, detoxification enzymes, etc.) responses (Tüzün & Stoks, 2020; Dinh Van et al., 2016). So far, this was beyond the aim of our study.

## 5 CONCLUSIONS, POTENTIAL IMPLICATIONS AND FUTURE DIRECTIONS

We showed evolved chlorpyrifos resistance in urban water flea populations, similar to observations in one other nontarget organisms (damselflies, Tüzün et al., 2015). These results corroborate the evidence that aquatic nontarget organisms are likely adapting to pesticide pollution in urban areas, which enters ponds via surface run‐off and aerial depositions. While we only tested resistance to chlorpyrifos, it is likely urban water fleas adapt to pesticides of the organophosphate and carbamate group (commonly applied in municipal and business districts), given they have similar modes of action and cross‐tolerance to different pesticides and other pollutants has been demonstrated in water fleas, wood frogs and fish (Bendis & Relyea, 2016a; Hua et al., 2014; Oziolor et al., 2016). Such evolved resistance to multiple pesticides, across multiple organisms in the aquatic food web, could significantly modulate genetic and species diversity in urban ponds, as well as associated ecosystem functions. For example, evolved chlorpyrifos resistance in *Daphnia pulex* prevented the development of a noxious algal bloom and impacted amphibian survivorship (Bendis & Relyea, 2016b). Although impacts of field exposures to pesticides seem predictable from ecotoxicological laboratory exposures (van der Hoeven & Gerritsen, 1997), future research on toxicant resistance in urban freshwater communities should specifically include targeted transplant experiments of focal species and communities (Brans et al., 2020). This enables to assess *in situ* to what extent evolution in multiple species in response to a multi‐stressor (warming, pollution, disturbance) anthropogenic environment leads to costs of adaptation or can ultimately stabilize ecosystem properties (water clarity, biodiversity), which will feed back to society via socio‐eco‐evolutionary feedbacks (Des Roches et al., 2020). Finally, we propose to test the impact of new pesticides allowed in organic agriculture, such as pyrethrins and pyrethroids, on nontarget species, as these pesticides are increasingly popular (also in community gardening efforts in cities) and a transition from synthetic conventional pesticides to pesticides of more natural origins is currently promoted under the current EU Common Agricultural Policy (2014–2020).

## CONFLICT OF INTEREST

The authors declare no conflict of interest.

## Supporting information

Supplementary Material

## Data Availability

R script and data are currently available on GitHub (https://github.com/MFajgenblat/brglmm‐chlorpyrifos). Data are additionally stored on Dryad.
