# Supplementary material for: Genetic differentiation in pesticide resistance between urban and rural populations of a nontarget freshwater keystone interactor, Daphnia magna
Source: Evol Appl. 2021 Oct 1;14(10):2541–52. doi: 10.1111/eva.13293 (PMC8549624; doi:10.1111/eva.13293)
Supplement: Supplementary file 1 — Supplementary Material [file EVA-14-2541-s001.pdf]

Supplementary Information ‘Genetic differentiation in pesticide resistance between urban and rural populations of a non-target keystone interactor, *Daphnia magna*’

MATERIAL AND METHODS

Study populations

Table S1. Locations of the *D. magna* study populations including the percentage built-up area (BA) in a radius of 3200 m around each pond, the according urbanization categorization.

Population		Location		Urbanization	
Pond	Code	Latitude (N)	Longitude (E)	%BA	Cat.
Brussel	*Brxl*	50.855464	4.347334	40.44	urban
Gent (Citadelpark)	*GenC*	51.0389	3.723744	24.242	urban
Gent (Muinkpark)	*GenM*	51.04251	3.731611	25.736	urban
Geraardsbergen	*Gera*	50.7842	3.915978	4.382	rural
Kortrijk (Blauwe Poort)	*BppK*	50.81624	3.271563	15.356	urban
Lapscheure	*Laps*	51.28253	3.355467	0.637	rural
Mechelen (Kruidtuin)	*Mech*	51.02402	4.484039	14.125	urban
Meerdaal (Zoete Waters)	*ZwMe*	50.82274	4.653691	2.017	rural
Midden-Limburg (BKN1)	*MidL*	50.98233	5.317858	3.721	rural
Damme	*Damm*	51.26207	3.276039	1.126	rural

Statistical analyses and results

<u>*Assessing MCMC convergence*</u>

Traceplots indicate excellent mixing of the chains for all model parameters (Figures S1-S5). For the standardized population- and clone-level random effects, all parameters were assessed but only random subsets of 8 out of respectively 10 and 49 parameters are visually presented. The Potential Scale Reduction Factor $\hat{R}$ did not reveal a lack of convergence either. The highest observed $\hat{R}$-value among all parameters is 1.0014. In addition, the lowest

effective sample size was 2,297.51. No divergent transitions were produced during the Hamiltonian Monte Carlo procedure.

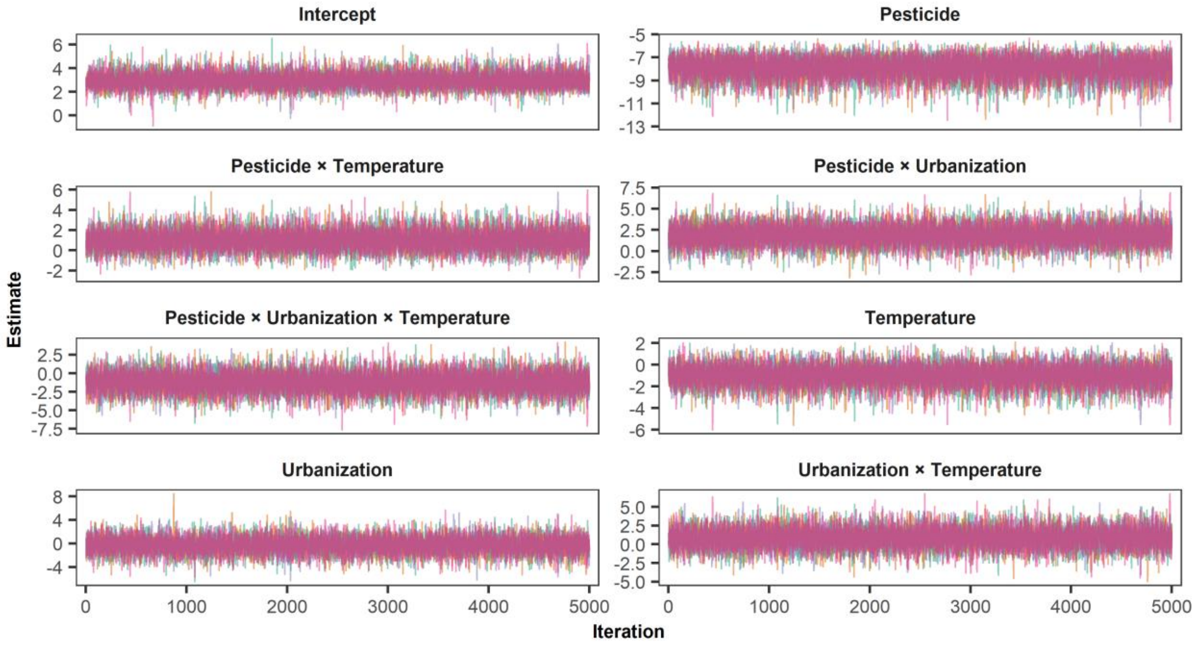

Figure S1. Traceplots for the regression parameters $\boldsymbol{\beta}$**,** for all iterations after the warmup stage. Each MCMC chain is represented by a different color.

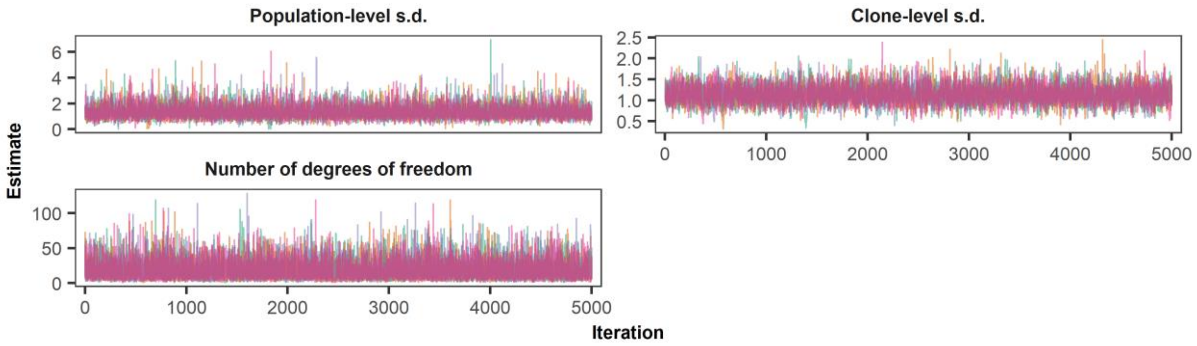

Figure S2. Traceplots for the population- and clone level standard deviations σ_{pop} and σ_{clone}, and the number of degrees of freedom ν of the Student's t-distributed clone-level random effects, for all iterations after the warmup stage. Each MCMC chain is represented by a different color.

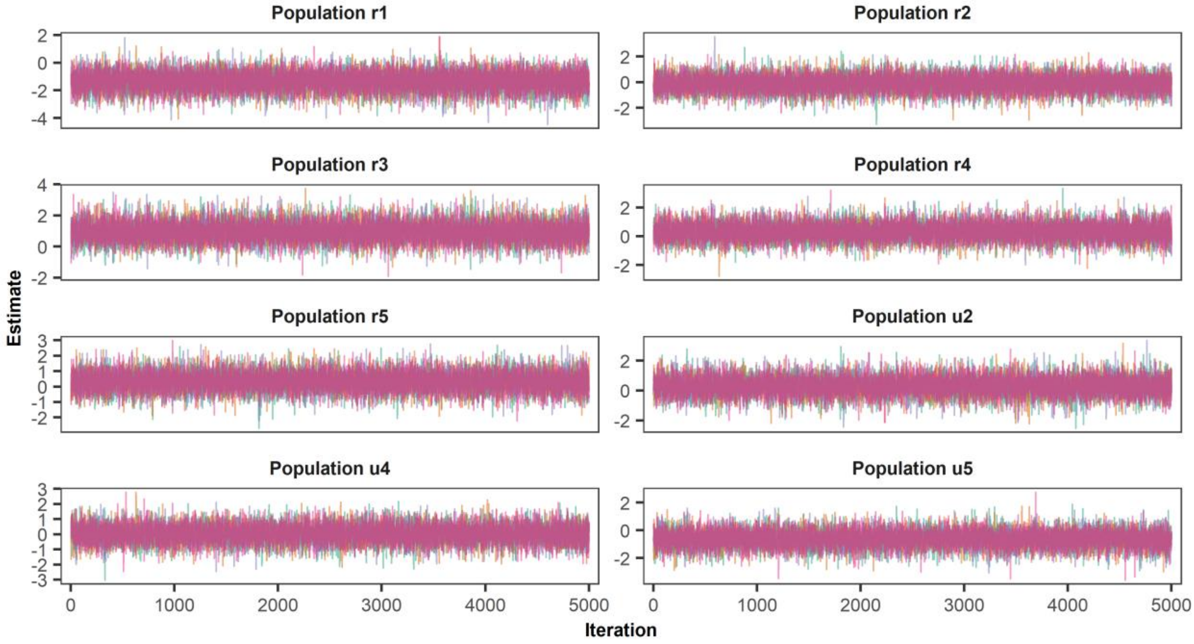

Figure S3. Traceplots for the standardized population-level random effects**,** for all iterations after the warmup stage. Each MCMC chain is represented by a different color.

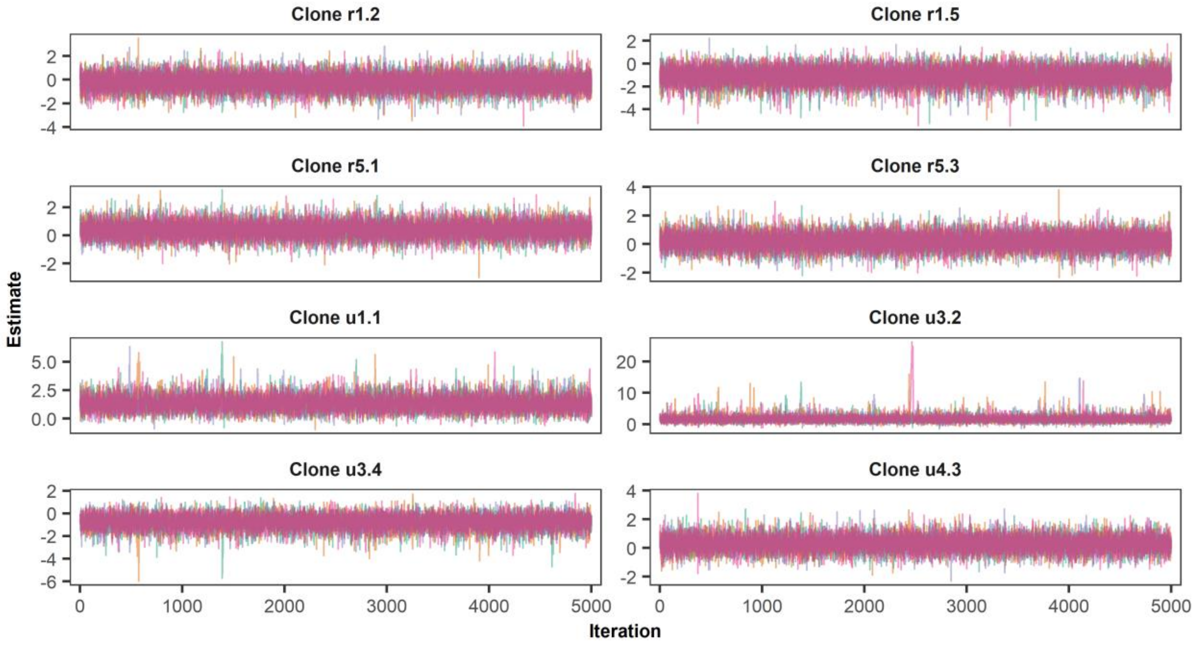

Figure S4. Traceplots for the standardized clone-level random effects**,** for all iterations after the warmup stage. Each MCMC chain is represented by a different color.

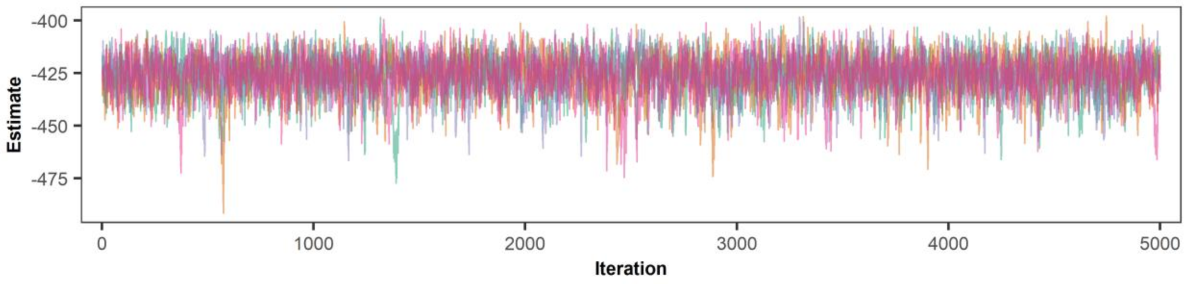

Figure S5. Traceplots for the model's log probability (up to an additive constant)**,** for all iterations after the warmup stage. Each MCMC chain is represented by a different color.

Prior sensitivity analyses

As indicated in the main text, we used following prior distributions for the model parameters:

$$\boldsymbol{\beta} \sim \mathrm{StudentT}(3,0,5),$$

$$\sigma_{pop}, \sigma_{clone} \sim \mathrm{StudentT}^{+}(3,0,5),$$

$$\nu \sim \mathrm{Gamma}(2,0.1).$$

Following Stan conventions, the Gamma distribution is here parametrized by the shape and rate (i.e. inverse scale) parameters.

We performed prior sensitivity analyses to assess the sensitivity of the posterior inferences to the prior specification. We consider three main scenarios (A, B and C) for the sensitivity analyses. In scenario A, we alter each (group of) parameter(s) separately, in a sequential fashion: $\boldsymbol{\beta}$ in subscenario A1, σ_{pop} in A2 and σ_{clone} in A3. For each (sub)scenario, we consider a strongly regularizing prior, where the scale parameters are divided by 5:

$$\boldsymbol{\beta} \sim \mathrm{StudentT}(3,0,1),$$

$$\sigma_{pop}, \sigma_{clone} \sim \mathrm{StudentT}^{+}(3,0,1),$$

a modestly regularizing prior choice where the scale parameters are halved:

$$\boldsymbol{\beta} \sim \mathrm{StudentT}(3,0,2.5),$$

$$\sigma_{pop}, \sigma_{clone} \sim \mathrm{StudentT}^{+}(3,0,2.5),$$

a vaguer prior choice where the scale parameters are doubled:

$$\boldsymbol{\beta} \sim \mathrm{StudentT}(3,0,10),$$

$$\sigma_{pop}, \sigma_{clone} \sim \text{StudentT}^{+}(3,0,10),$$

and a strongly vaguer prior choice where the scale parameters are multiplied by 5:

$$\boldsymbol{\beta} \sim \text{StudentT}(3,0,25),$$

$$\sigma_{pop}, \sigma_{clone} \sim \text{StudentT}^{+}(3,0,25).$$

In scenario B, we specifically investigate prior choice for the number of degrees of freedom of the Student's t-distributed clone-level random effects, where we evaluate an alternative prior choice that places more prior mass towards lower values of ν:

$$\nu \sim \text{Gamma}(2,0.5),$$

and an alternative prior that is wider than the original choice:

$$\nu \sim \text{Gamma}(3,0.05).$$

In scenario C, we simultaneously alter the prior choice for all parameters $\boldsymbol{\beta}, \sigma_{pop}$ and σ_{clone}, except for ν. Here too, we consider the same strongly regularizing priors, regularizing priors, vaguer priors and strongly vaguer priors as outlined for scenarios A1-A3.

For each scenario (A, B1, B2, B3 and C), we refit the model with the alternative prior specifications and plot and compare the obtained posterior densities with these of the original model. We use 4 chains of 2,000 iterations each (of which 1,000 are discarded as warmup) instead of 10,000, as this offers a major speedup without affecting MCMC convergence (due to excellent mixing). Specifically, we monitor changes with respect to the posterior densities of $\boldsymbol{\beta}, \sigma_{pop}, \sigma_{clone}, \nu$ and the log probability (up to an additive constant).

The prior sensitivity analyses do not reveal evidence that the presented posterior inference is particularly sensitive to the prior specification (Figures S6-S10). Alternative prior

specifications for the regression parameters have the strongest influence on posterior inference, though differences are relatively modest. The two key effects discussed in the manuscript (main effect of pesticide exposure and its interaction with an urban origin) maintain a high probability of a respectively negative and positive effect under all evaluated scenarios, even under a strongly regularizing prior with unit variance. In some cases, the strongly vaguer prior specification causes the Hamiltonian Monte Carlo procedure to produce a few divergent transition: too vague priors are known to frustrate Hamiltonian Monte Carlo.

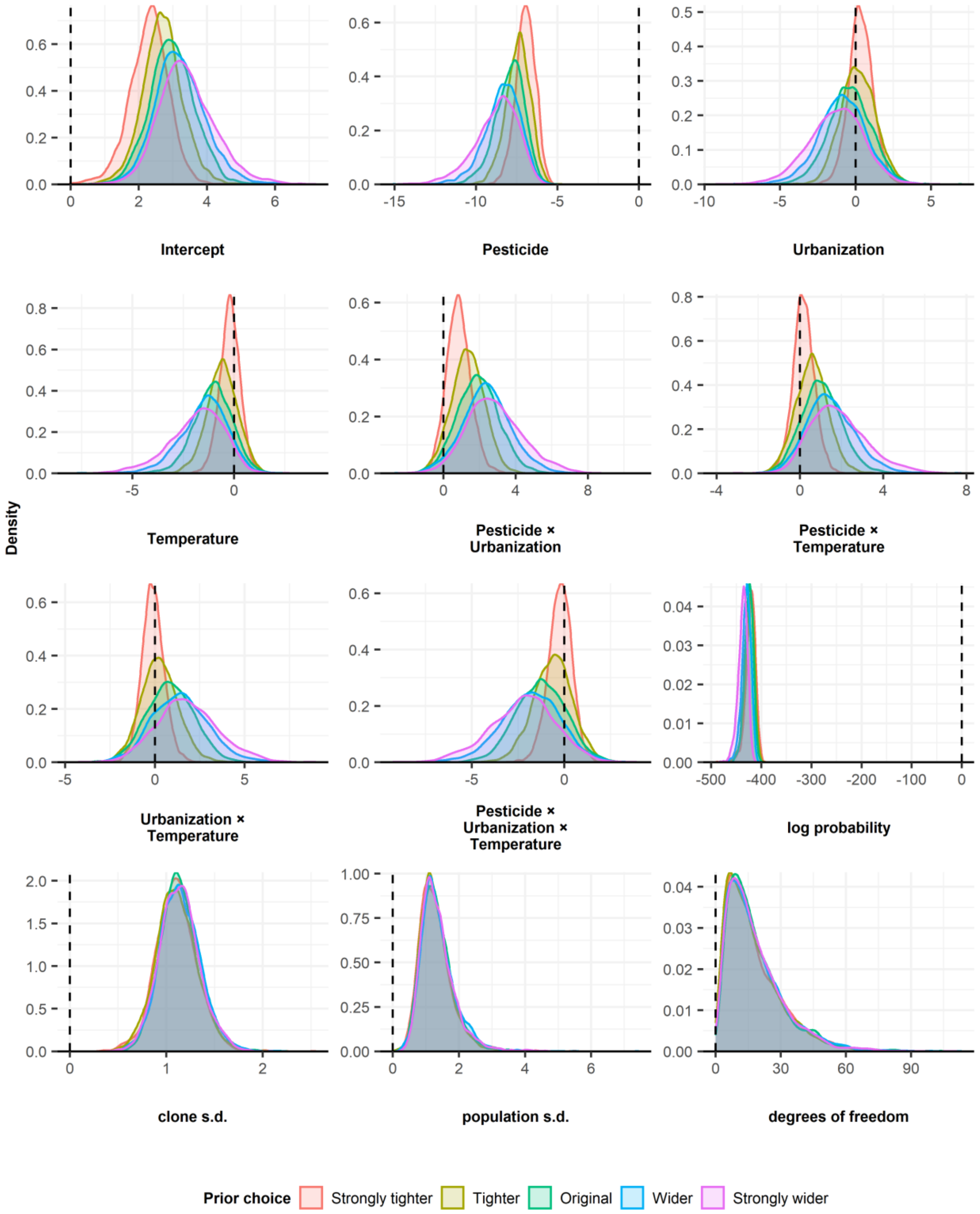

Figure S6. Prior sensitivity analysis for scenario A1, where alternative prior specifications are evaluated for the regression parameters. Each colored density represents the posterior distribution obtained using a specific prior choice. The vertical dashed line represents a zero-effect.

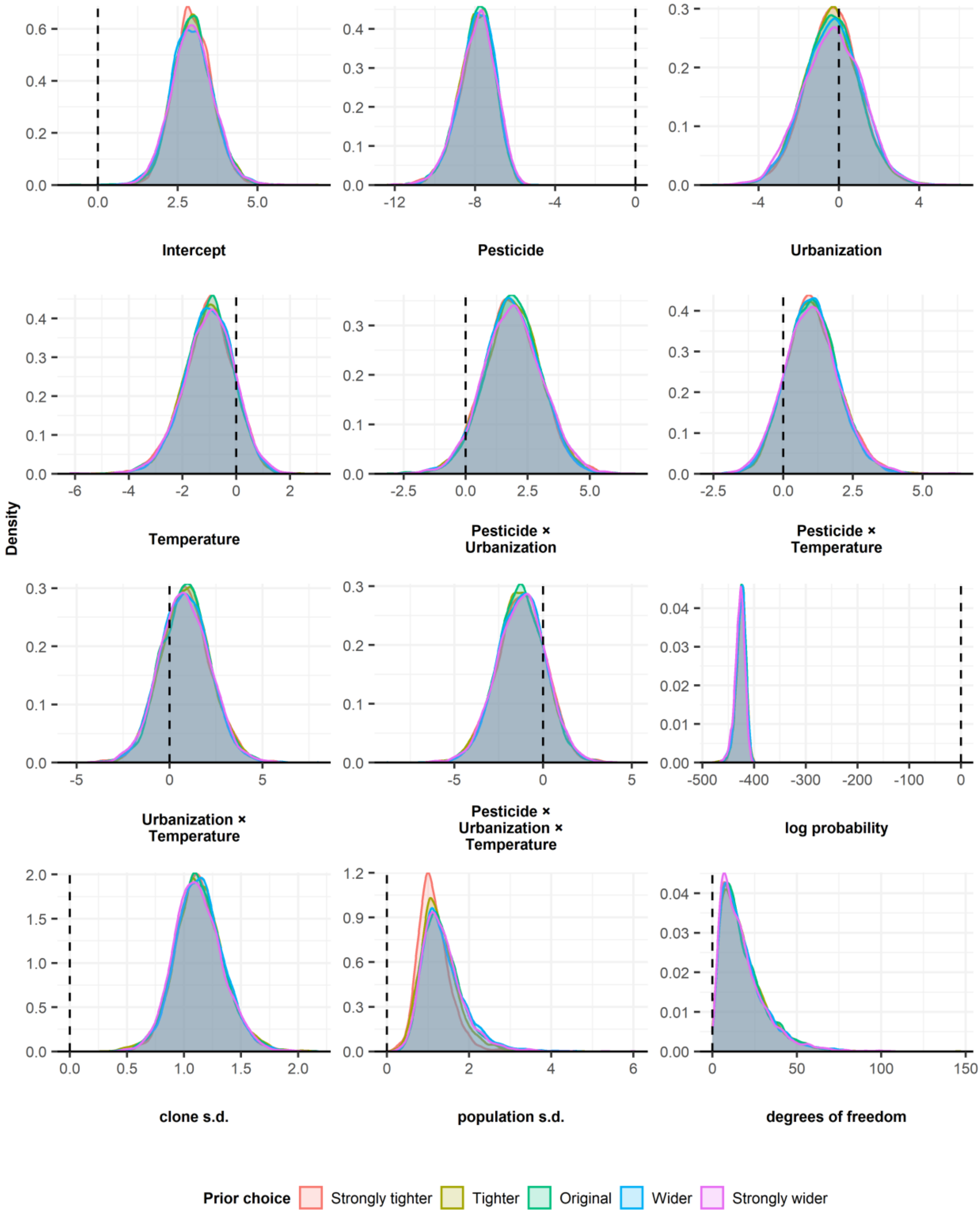

Figure S7. Prior sensitivity analysis for scenario A2, where alternative prior specifications are evaluated for the population-level standard deviation. Each colored density represents the posterior distribution obtained using a specific prior choice. The vertical dashed line represents a zero-effect.

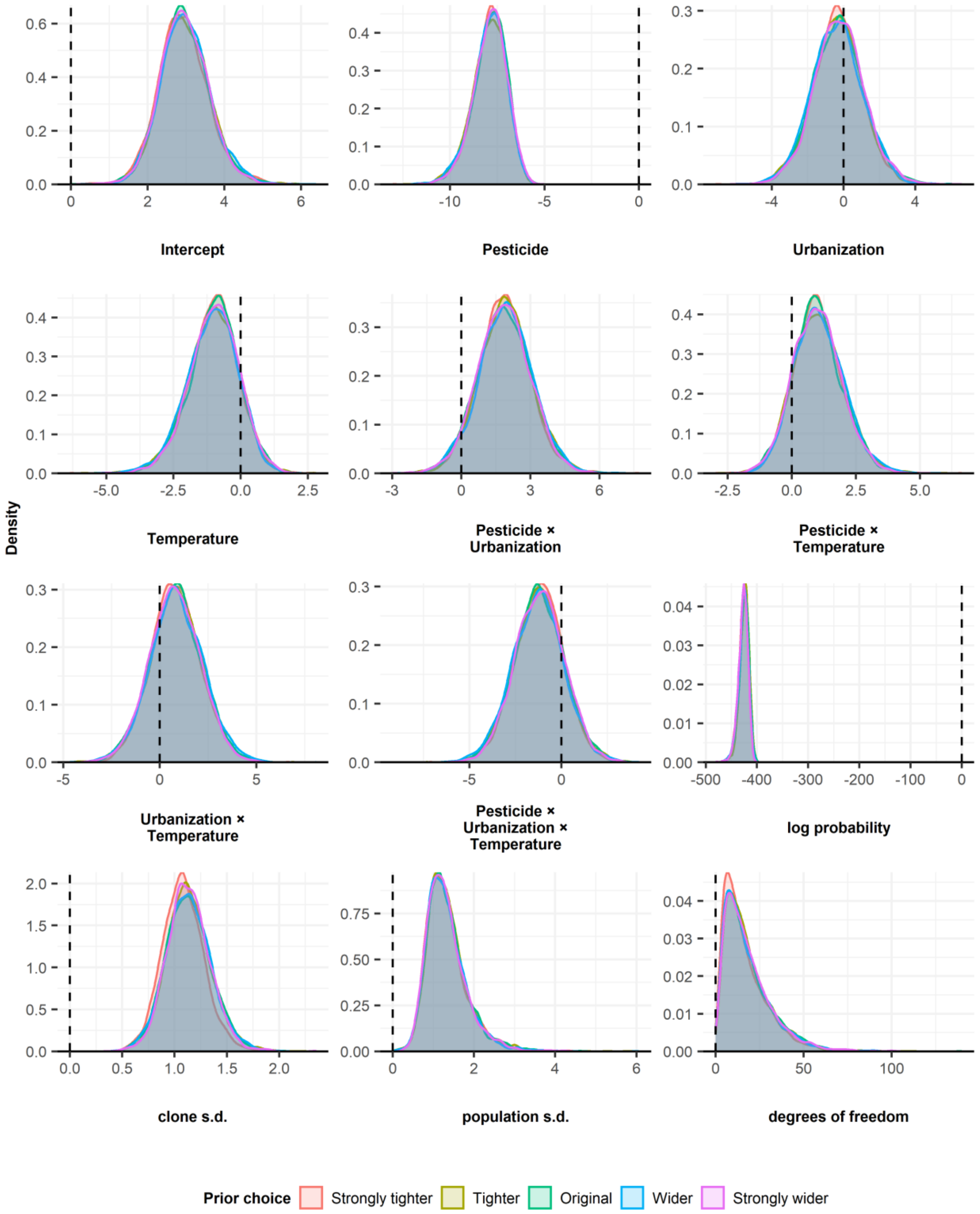

Figure S8. Prior sensitivity analysis for scenario A3, where alternative prior specifications are evaluated for the clone-level standard deviation. Each colored density represents the posterior distribution obtained using a specific prior choice. The vertical dashed line represents a zero-effect.

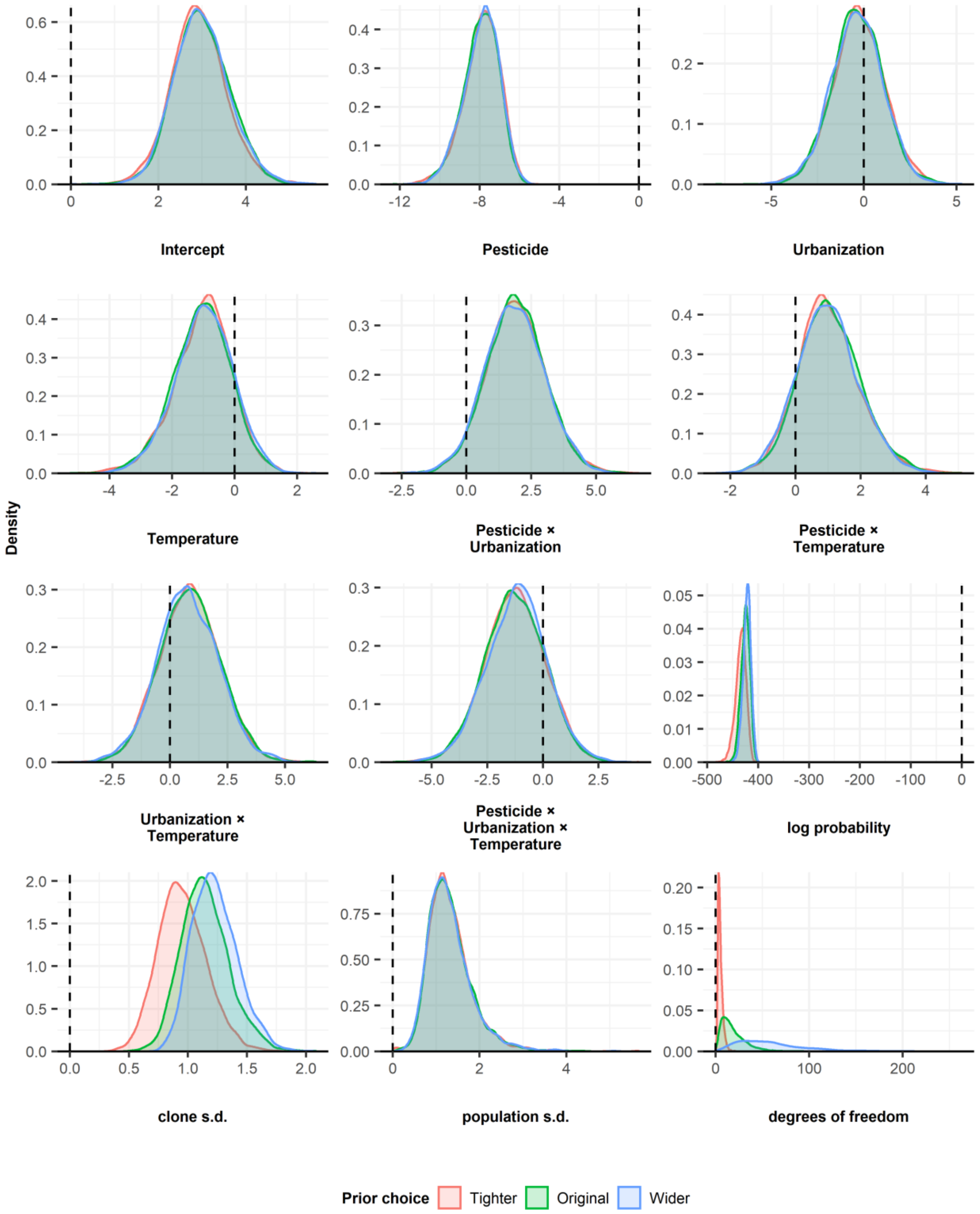

Figure S9. Prior sensitivity analysis for scenario B, where alternative prior specifications are evaluated for the number of degrees of freedom of the Student's *t*-distributed clone-level random effects. Each colored density represents the posterior distribution obtained using a specific prior choice. The vertical dashed line represents a zero-effect.

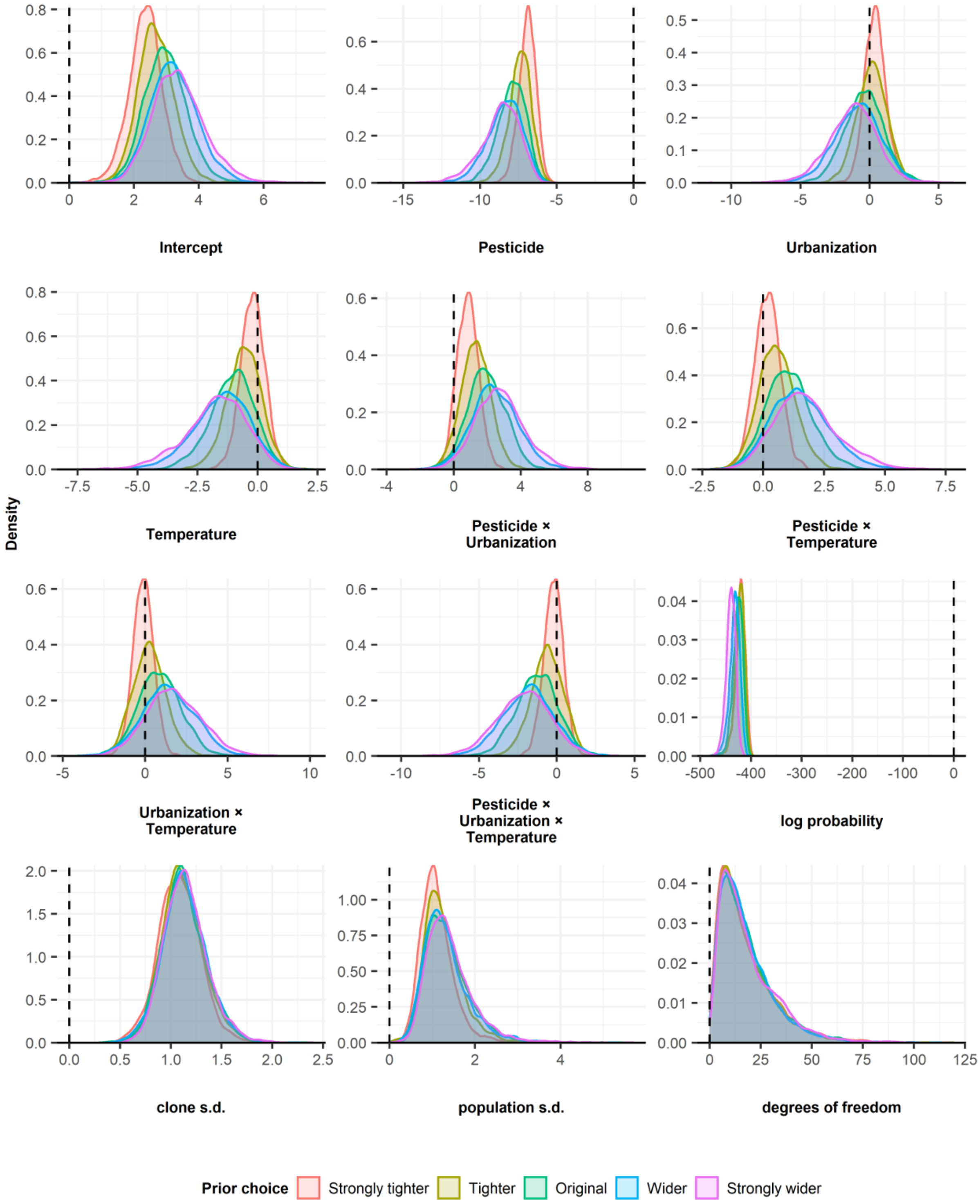

Figure S10. Prior sensitivity analysis for scenario C, where alternative prior specifications are evaluated for all parameters (except the number of degrees of freedom) simultaneously. Each colored density represents the posterior distribution obtained using a specific prior choice. The vertical dashed line represents a zero-effect.

Posterior predictive checking

We asses goodness-of-fit by means of a simple graphical posterior predictive check. For this, we sample 20,000 draws (4 chains x 5,000 post-warmup draws) from the posterior predictive distribution, as part of the MCMC procedure. We then compare the distribution of frequencies of the number of surviving individuals with the observed frequency of the number of surviving individuals. This graphical posterior predictive check does not reveal strong deviations of the posterior predictive distribution compared to the observed data (Figure S11). However, the model does underpredict the number of replicates with low and high survival to a moderate extent (mainly for instances with 0 and 5 surviving individuals). Hence, there seems to be some overdispersion with respect to the considered binomial distribution.

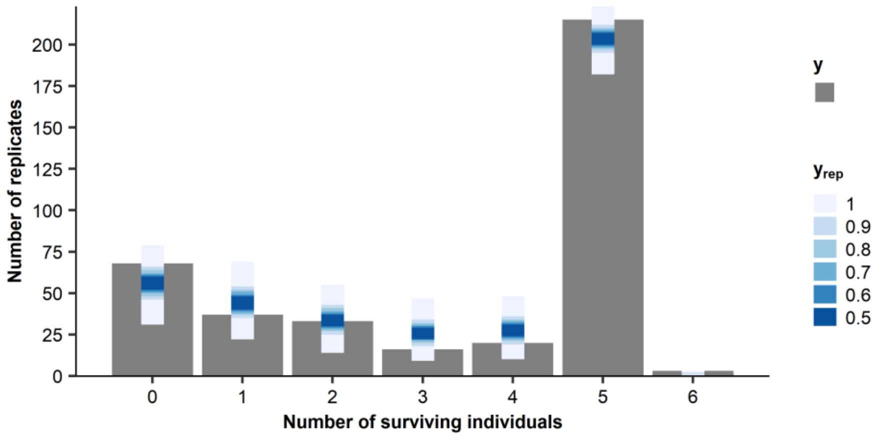

Figure S11. Graphical posterior predictive check. The grey bar plot describes the frequencies of the number of surviving individuals per replicate. The blue interval plots represent the posterior predictive distribution for these counts.

In order to ascertain that our findings are robust against the presence of overdispersion, we developed a second version of the model that includes an observation-level random effect (which has a similar effect as using a beta-binomial distribution). Stan code for this model is also available online ("Overdispersion_model.stan" on https://github.com/mfajgenblat/brglmm-chlorpyrifos) and can completely be run from the original R-script after changing the model file name argument in the stan()-function.

Graphical posterior predictive checks indicate that his second model is able to accommodate overdispersion (Figure S12). Compared to the original model, this extended model yields close to identical inferences (Figure S13).

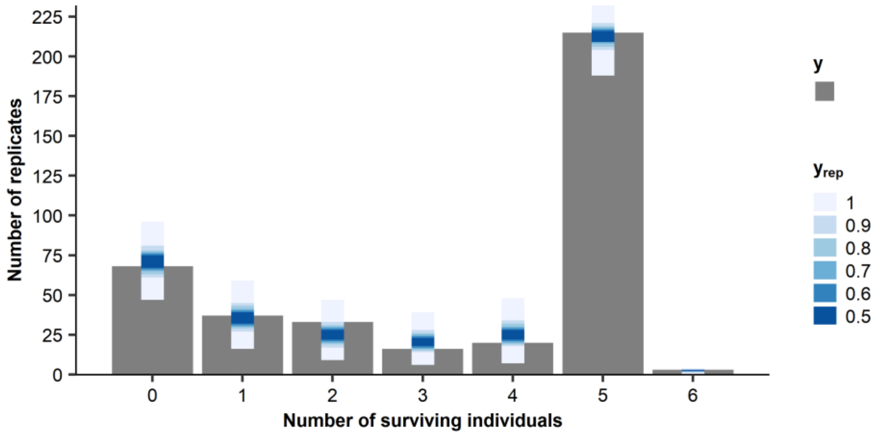

Figure S12. Graphical posterior predictive check for the extended model that accommodates overdispersion. The grey bar plot describes the frequencies of the number of surviving individuals per replicate. The blue interval plots represent the posterior predictive distribution for these counts.

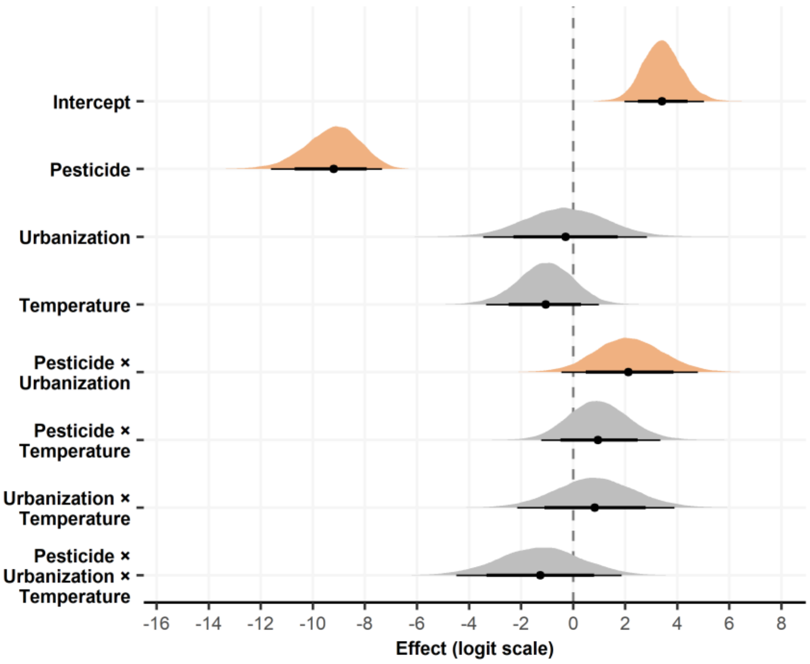

Figure S13. Estimated main and interaction effects (logit scale) using the extended model that accommodates overdispersion. Bayesian posterior densities are represented by density plots, 80 and 95% credible intervals by horizontal lines and posterior medians by black dots. Variables for which the posterior probability that their effect is either positive or negative exceeds 0.95 (given model and data), are shown in orange.

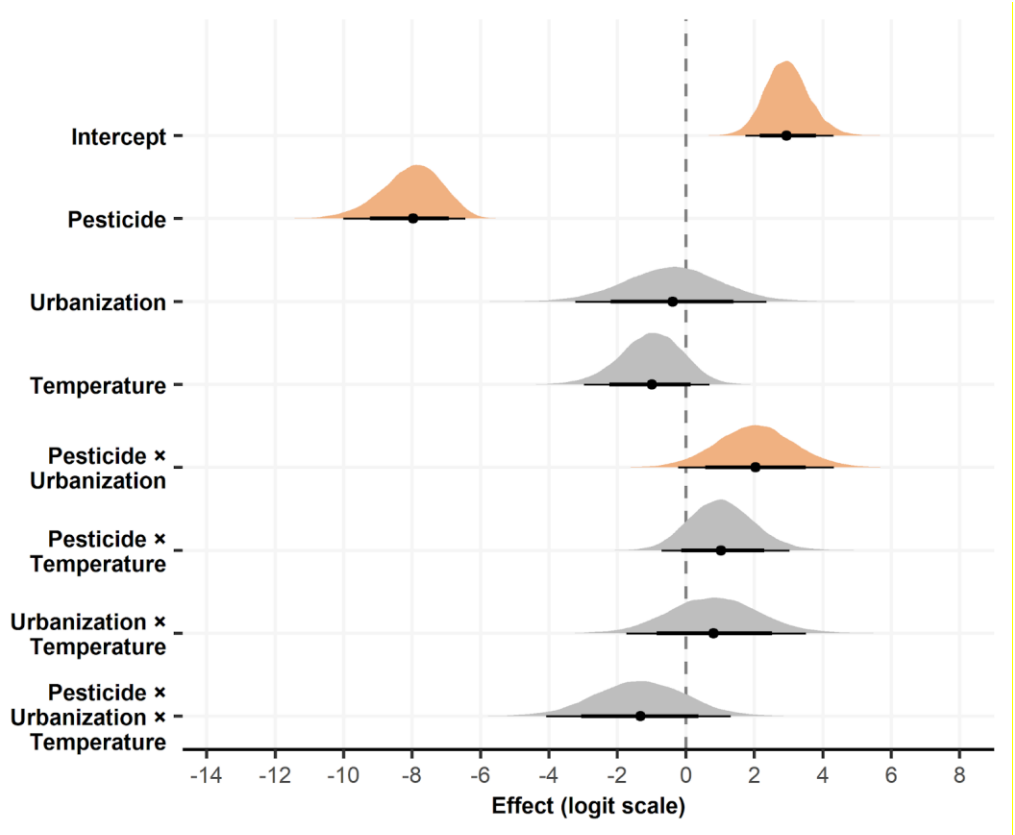

Figure S14. Estimated main and interaction effects on survival (logit scale) obtained from an adjusted analysis in which the 4 units that contained 6 animals were omitted from the data.

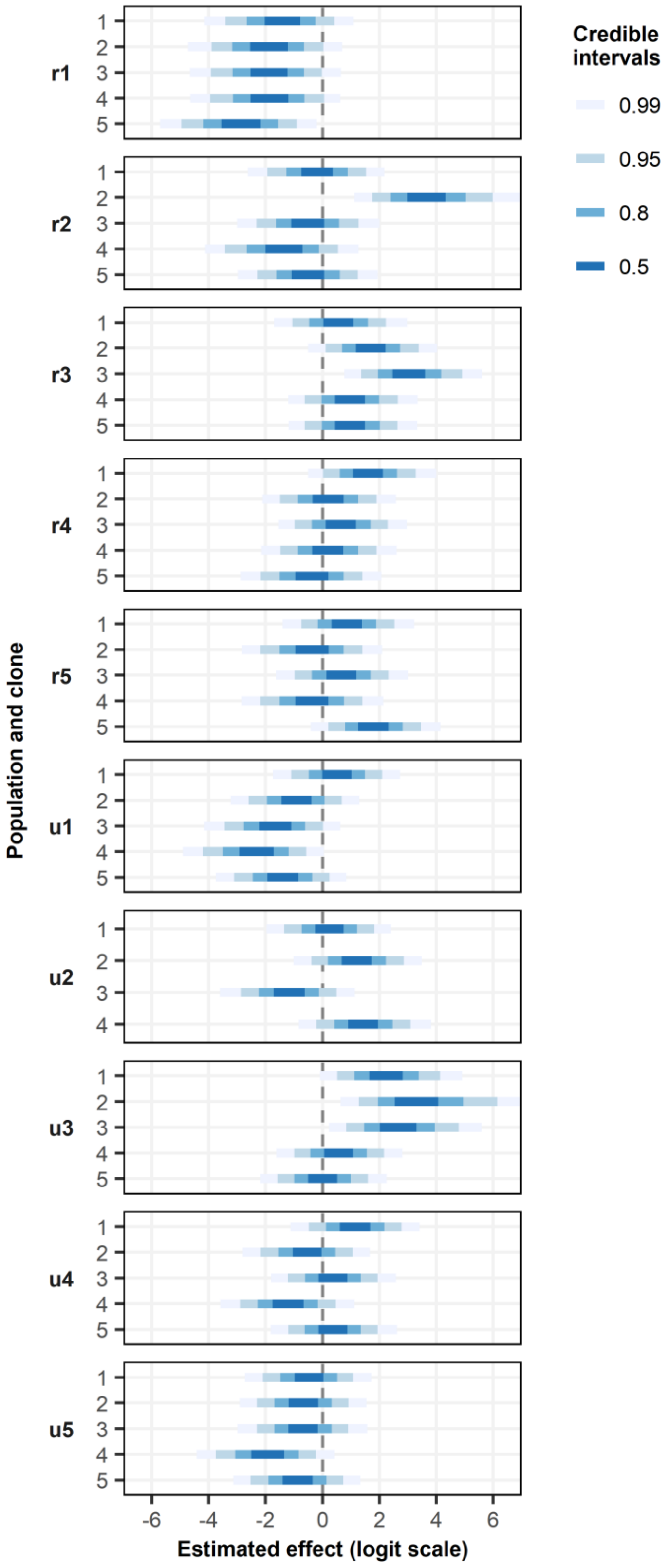

Figure S15. Summarized posterior distributions of the clone- and population-specific random effects, showing the heterogeneity with respect to chlorpyrifos sensitivity among populations and clones. The

pure population-level effects are not shown explicitly, but are implicitly included by showing the sum of the population- and clone-level random effects. The colored lines represent 50, 80, 95 and 99% credible intervals. Negative values correspond to a decreased survival probability, while positive values correspond to an increased survival probability, relative to the survival in the average condition, population and clone
